# Intratarget Microdosing for Deep Phenotyping of Multiple Drug Effects in the Live Brain

**DOI:** 10.3389/fbioe.2022.855755

**Published:** 2022-03-18

**Authors:** Jennifer Kim, Sebastian W. Ahn, Kyle Deans, Devon Thompson, Benjamin Ferland, Prajan Divakar, Christine Dominas, Oliver Jonas

**Affiliations:** ^1^ Department of Radiology, Brigham and Women’s Hospital, Harvard Medical School, Boston, MA, United States; ^2^ Nanostring Technologies, Inc., Seattle, WA, United States

**Keywords:** microdevice, polymer formulation, Alzheimer’s disease, drug sensitivity, *in vivo*, high-throughput screen

## Abstract

A main impediment to effective development of new therapeutics for central nervous system disorders, and for the *in vivo* testing of biological hypotheses in the brain, is the ability to rapidly measure the effect of novel agents and treatment combinations on the pathophysiology of native brain tissue. We have developed a miniaturized implantable microdevice (IMD) platform, optimized for direct stereotactic insertion into the brain, which enables the simultaneous measurement of multiple drug effects on the native brain tissue *in situ*. The IMD contains individual reservoirs which release microdoses of single agents or combinations into confined regions of the brain, with subsequent spatial analysis of phenotypic, transcriptomic or metabolomic effects. Using murine models of Alzheimer’s disease (AD), we demonstrate that microdoses of various approved and investigational CNS drugs released from the IMD within a local brain region exhibit *in situ* phenotypes indicative of therapeutic responses, such as neuroprotection, reduction of hyperphosphorylation, immune cell modulation, and anti-inflammatory effects. We also show that local treatments with drugs affecting metabolism provide evidence for regulation of metabolite profiles and immune cell function in hMAPT AD mice. The platform should prove useful in facilitating the rapid testing of pharmacological or biological treatment hypotheses directly within native brain tissues (of various animal models and in patients) and help to confirm on-target effects, *in situ* pharmacodynamics and drug-induced microenvironment remodeling, much more efficiently than currently feasible.

## Introduction

There is a high unmet need in neuroscience and the treatment of central nervous system (CNS) disorders to rapidly understand the efficacy of potential treatments, tool compounds or probes on the native brain (Butcher, 2007; Mangialasche et al., 2010; Cummings, 2011; Anderson et al., 2017).

Current *in vitro* models provide a high-throughput approach to performing such measurements, but their fundamental limitation is that critical aspects of CNS pa thophysiology are not faithfully recreated outside of the organism, where the interaction of numerous specific cell types and distinct tissue architecture are key determinants of physiological function and therapy response (Holloway et al., 2021). While such systems are useful for understanding mechanisms of basic interaction between subsets of cell types such as astrocytes and microglia or neurons and oligodendrocytes (Abud et al., 2017; Timmerman et al., 2018), they are unable to recapitulate the complex interaction of the many cell types present in the intact brain, and have thus provided little actionable insight into expected *in vivo* responses to drug treatments (Nikolakopoulou et al., 2021).

One area of high translational interest is the interaction of brain cells with immune cells, as this is of central importance to the study of neurodegenerative disorders. But due to the complexity of interactions among a variety of cell types, and the role that brain architecture plays (for example in the migration of activated T-cells), *in vitro* studies are fundamentally limited in recreating such interactions accurately (Abdeen et al., 2016; Amit et al., 2016; Dong et al., 2019). Another area of high interest is the role played by metabolic networks in the regulation, growth, and activation of various cell types in the brain. Metabolites serve critical functions as both nutrients and signaling molecules, and their relative abundance depends heavily on the interplay of all cell types that comprise the microenvironment of the brain (Vanhanen et al., 2006; Kim et al., 2014; Varma et al., 2018).

Traditional systemic dosing studies of drugs or combinations remain the gold standard in assessing efficacy for drug translation but are often challenging to conduct because of well-documented difficulties related to penetration of the blood brain barrier (Zenaro et al., 2017; Mulvihill et al., 2020). This is particularly true for novel drugs and experimental agents. Such studies may require significant effort to optimize the pharmacokinetic (PK) and toxicological properties of a compound, which hinders rapid testing of its efficacy *in vivo* (Piton et al., 2018; Cummings et al., 2019; Cummings et al., 20192019). During the early lead identification and target validation phases of drug development, large systemic screens involving many compounds and combinations are often cost prohibitive, especially in light of the PK challenges.

The dichotomy between *in vitro* models that fail to replicate the complexity of brain physiology, and systemic studies with low-throughput and challenging PK issues, may be overcome by using an intra-target microdosing approach for *in situ* parallel testing of multiple agents.

Here we describe a platform to measure the effect of up to 16 agents directly within the native brain of mice. The approach uses IMDs (Jonas et al., 2015; Davidson et al., 2016) that are inserted into the brain using minimally invasive biopsy and release microdoses of different agents into defined and spatially separate regions of the brain, in a time and concentration dependent tunable fashion. A variety of spatial analysis techniques, such as spatial proteomics of 56 markers, are employed to characterize native tissue responses to each of the 16 drugs in a single mouse brain. These spatial drug effect measurements are overlayed with drug release profiles to measure local PK/PD using a variety of cell-type specific markers in a concentration dependent manner, and describe the effect of each drug on neurons, astrocytes, microglia, and oligodendrocytes, as well as immune cells and macrophages in the intact brain.

We apply the platform to a case study involving multiple common and experimental treatments in murine models of Alzheimer’s disease (AD). The approach allowed us to determine *in vivo* response of 12 pharmacological agents on key physiological parameters of the AD phenotype such as expression of various tau isoforms and inflammation markers.

The combination of multiplexed high-precision microdose drug release into targeted regions of the brain, along with spatially intact analysis of response phenotypes, provides a unique platform to examine the effects of multiple drugs on live brain pathophysiology which can complement traditional systemic and *in vitro* approaches. We envision three major applications for this platform for basic and translational researchers: one, to measure *in vivo* drug effects of novel compounds, probes or combinations, without the need for PK and toxicological optimization; two, to screen a large set of compounds or combination treatments *in vivo* to determine which pharmacological interventions provide the most favorable response phenotype for a given CNS disorder; and three, to enable a systems biology approach for comprehensive functional characterization of a disease state by performing multiple simultaneous biochemical perturbations on the brain of animal models, and potentially patients.

## Results

### Concept and Technical Workflow

**FIGURE 1:**
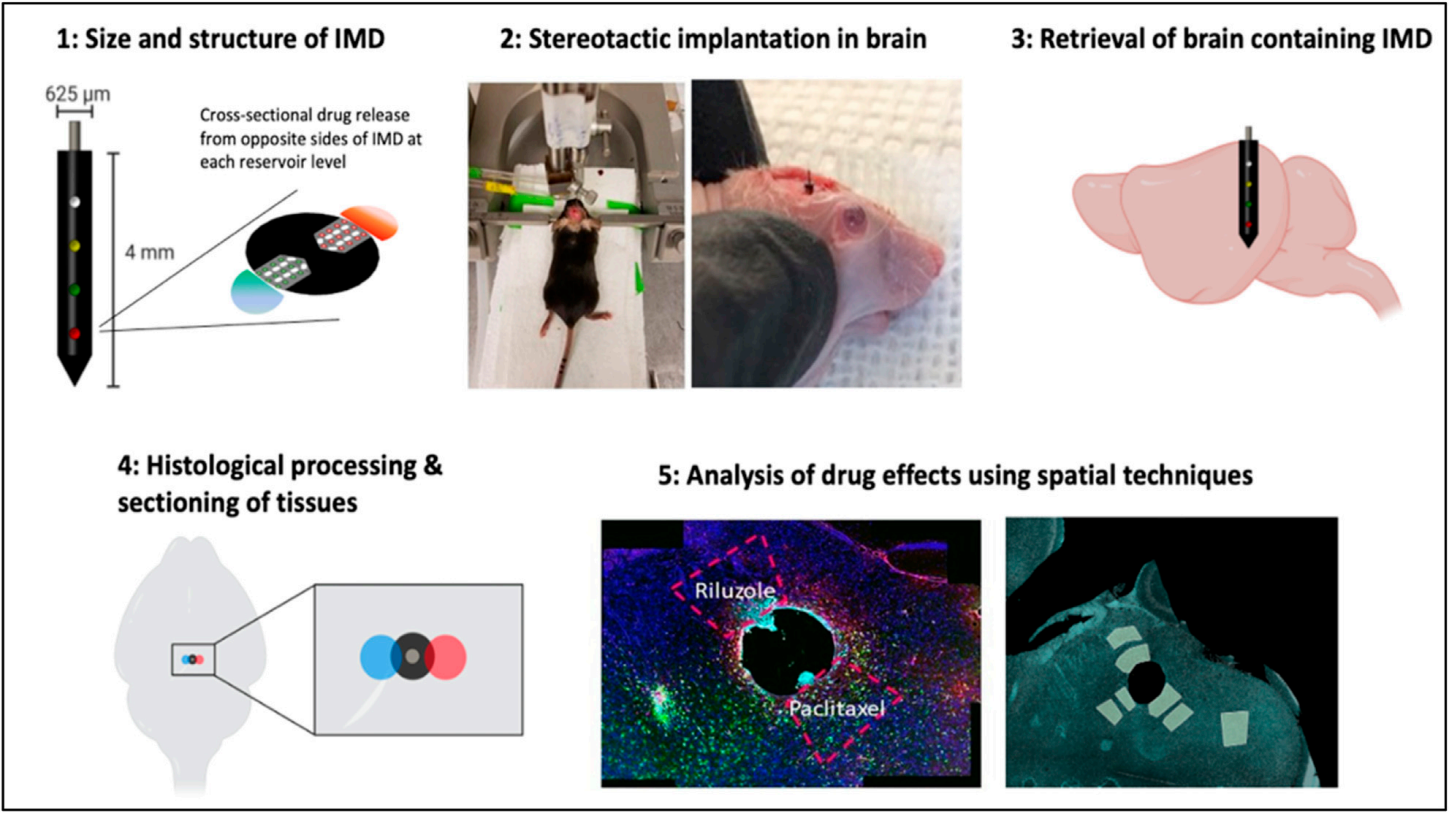
Technical workflow of IMD implantation, drug release, processing and analysis in murine brain.

Our platform consists of implantable microdevices (IMDs) placed into the brain to release multiple microdoses of individual drugs or combinations into spatially confined regions of the brain. The technical workflow is shown in Figure 1.

Upon stereotactic placement targeting a specific brain region, the IMD remains in the brain for several hours to days (for the purposes of the current study we demonstrate implantation times of 3 and 7 days, with placement into the hippocampal dentate gyrus). During this time, drugs from each reservoir are released into a region of tissue immediately adjacent to each reservoir. The spatial extent of drug release is controlled by the formulation of the drug in a polyethylene glycol (PEG) matrix. Each drug reacts with the brain tissue in its native context. The specimen containing the IMD and surrounding tissue is then retrieved, and follows a standard workflow for downstream analysis using histopathological, proteomic, and other analysis.

### Obtaining localized regions of microdose drug exposure into confined regions of the brain at desired concentrations

In order to obtain response phenotypes for multiple agents in a single mouse brain, it is critical that drug release from an individual reservoir is confined to spatially separate regions of the brain without overlap with other reservoirs.

**FIGURE 2:**
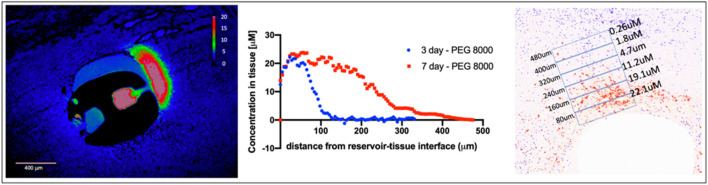
Precise measurement of drug concentration in the murine brain. (Left) Local drug release of MK2206 from IMD reservoir into thalamus, measured at t = 7d. (Middle) Drug concentration shown as a function of distance along the gradientrom center of IMD reservoir. (Right) Distance and concentration dependent drug effect to obtain intra-brain PK/PD.

To control the rate and concentration of drug release into brain tissue, drugs are formulated in a polyethylene glycol (PEG) polymer matrix before they are loaded into micro-reservoirs on the IMD. The molecular weight of PEG determines the rate of release of drug into tissue, and is precisely tunable to achieve a range of desired drug concentrations. Release into tissue occurs by passive diffusion over the course of several hours to days. For the current study, we demonstrate release times of 3 and 7 days. The total amount of drug in each reservoir is ∼2 ng, which is several orders of magnitude below systemic dosing levels for both mice and patients. Proper selection of PEG at the appropriate molecular weight and for a desired duration of implantation and ratio of drug to polymer allows a defined local drug concentration range to be achieved, as shown in Figure 2. Drug concentration has been shown to be controlled predominantly by polymer selection across a large set of small-molecule agents (Jonas et al., 2015). As shown in Figure 2, at t = 7d, the concentration range of 0.2–22 μM is demonstrated. Maximum concentrations up to ∼100 μM are achievable with this approach. Tissue concentrations below 0.2 μM are readily achievable by reducing the ratio of drug to polymer in a given microdevice reservoir. The maximal diffusion distance of ∼450 μm is significantly less than the inter-reservoir separation distance of 800 μm, thus ensuring that drugs from adjacent reservoirs do not overlap within a given region of interest.

### Spatially integrated measurement of intra-brain PK/PD and microenvironment remodeling for 16 drugs in a mouse brain

**FIGURE 3:**
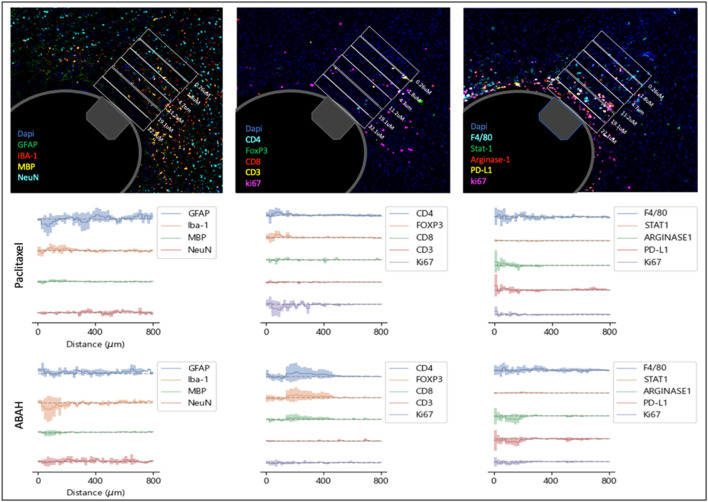
(Top) Multiplexed immunofluorescence analysis of distance-dependent drug effects using 13 cellular and phenotype markers. Rectangular boxes serve as distance markers of 90um width. Example is shown for paclitaxel at t = 7d in B6 hMAPT mice (Bottom) Spatial plots of marker expression indicating concentration-dependent drug effects, shown for paclitaxel and ABAH. Each plot is generated by subtracting the expression levels from control regions from the respective drug-exposed ROI. Lines represent the mean value from n = four to six mice (shaded area = SD).

Using the distance-dependent drug concentration gradients shown in Figure 2, we calculated the expression of 13 histological markers of drug effect on various cell types and phenotypes in the brain microenvironment, as a function of drug concentration in the brain tissue (Figure 3). Control regions were calculated from tissue regions directly adjacent to the IMD but with reservoirs containing no drug, in order to account for tissue responses caused by IMD insertion. In this manner, precise PK/PD curves are generated for all agents on the IMD across the 13 markers employed (Figure 3).

**FIGURE 4:**
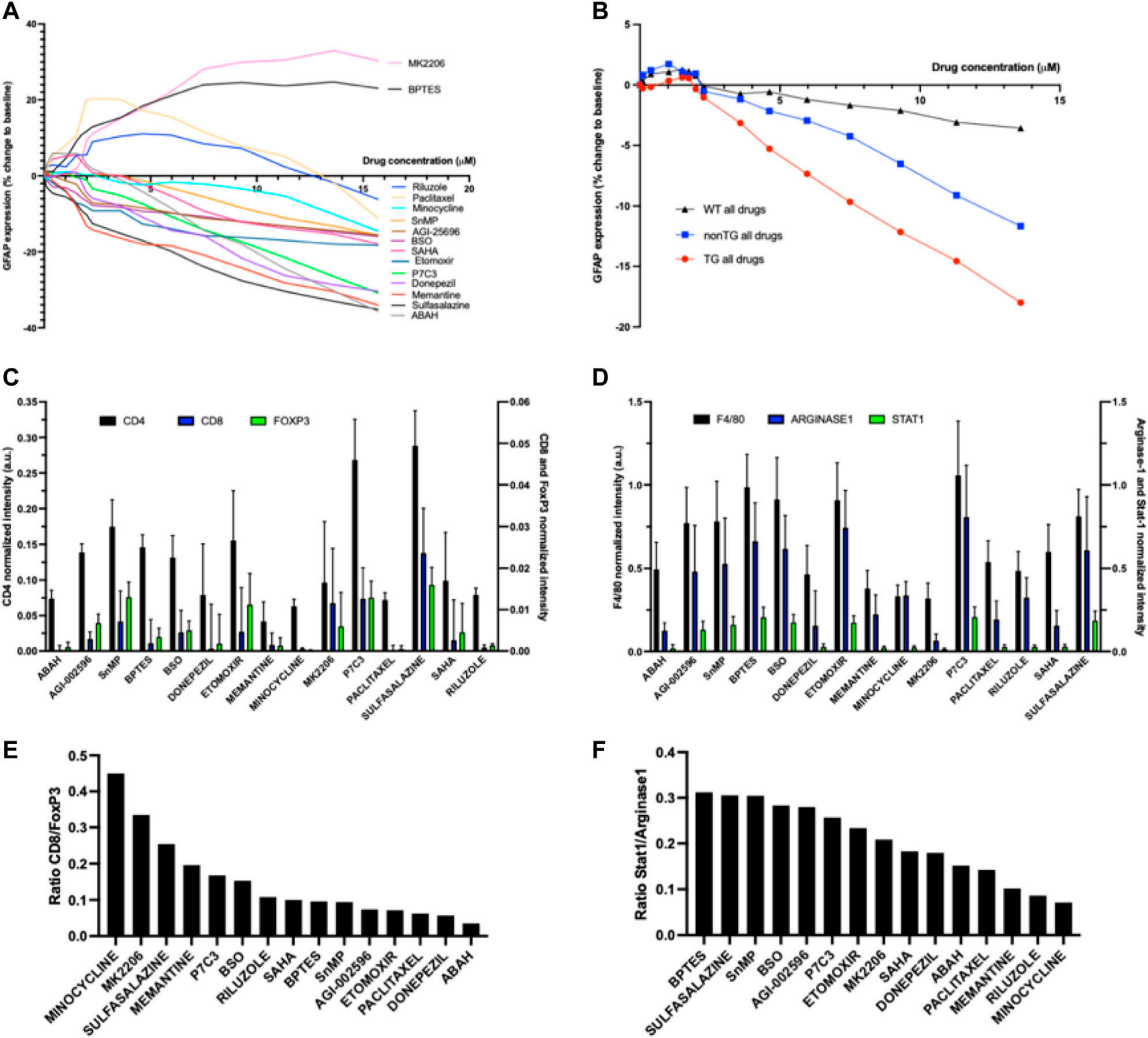
**(A)** Precise PK/PD curves GFAP expression versus drug concentration for 15 individual drugs at 7 days (n = four to six per drug) **(B)** PK/PD for GFAP expression as a function of concentration for all drugs combined, compared across three animal models **(C)** and **(D)** Quantitative description of commonly used T-cell and macrophage markers, respectively, per individual drug region (n = four to six per drug) **(E)** Ratio of CD8 to FoxP3 in each region of drug activity to measure effects of each drug’s effect on T-cell state **(F)** Ratio of Stat1 to Arginase-1 FoxP3 in each region of drug distribution to measure each drug’s effect on macrophage polarization.

We observe that drugs remodel the brain microenvironment in a concentration-dependent manner, with changes in marker expression exhibiting a distance-dependent effect across drugs. An example of a pathobiological phenotype that has been associated with favorable outcomes in AD is reduced levels of activated microglia, as these are associated with neuronal death, increased plaque formation and tau pathology (Colonna and Butovsky, 2017; Pearce and Pearce, 2017; Spangenberg and Green, 2017; Perea et al., 2020). Analogously, increases in astrocyte activation and astrogliosis have been linked to neurotoxicity, tau pathology, neuronal death and cognitive impairment (Liddelow et al., 2017; Chun et al., 2020). We determined which agents cause the greatest positive and negative changes in GFAP, a commonly marker of activated astrocytes, and define the intra-brain concentrations that are required to reach these effects (Figure 4A). Two of the 15 agents tested in the AD model, the AKT inhibitor MK2206 and the glutaminase inhibitor BPTES, led to an increase in GFAP expression which plateaus around 7 μM. Riluzole and Paclitaxel exhibit initial increase in GFAP at low concentrations, followed by decreases in GFAP upward of ∼4 μM. All other compounds effected a consistent, concentration-dependent decrease in GFAP expression, which is observed to be strongest for Memantine in the range of 1–5 μM, and for ABAH at maximum doses.

Analogously, we observe that local treatment with ABAH, a myeloperoxidase inhibitor, and Donepezil, an acetylcholinesterase inhibitor, lead to a reduction in expression of Iba-1, a marker for microglia, at concentrations of ∼5–8 μM, relative to control. ABAH also induces an increase in NeuN, a marker for neurons throughout the region of drug distribution at t = 7d. Among compounds targeting metabolism, P7C3, a pro-neurogenic and neuroprotective chemical that targets NAMPT enzyme, exhibits the highest degree of Iba-1 reduction and NeuN increase (Supplementary Figure S1).

We examined whether the effect of drugs was more pronounced in animal models that mimic AD pathobiology. This was assessed by comparing the effects of all compounds tested across the TG, non-TG and wild type mice. We observed that the set of drugs examined in this study showed the highest pharmacodynamic effect in GFAP reduction in the TG model, followed by non-TG and the wild type mouse (Figure 4B), across the entire concentration range. The effect was observed to be strongest at doses greater than 2 μM.

### Effects on immune modulation and anti-inflammatory effects

A question of high interest to researchers is which therapeutic interventions are capable of stimulating an anti-inflammatory immune response in the brain, and relatedly, whether metabolic interventions such as those from the gut microbiome, are capable of inducing such immune responses (Varma et al., 2018; Singh et al., 2021). The IMD approach is capable of identifying which agents among the range of therapies tested, induces the most favorable immune responses, specifically the induction of defined T-cell and macrophage phenotypes, and determine the exact local concentration range for which each agent exhibits its maximal effect.

Among the agents targeting specific metabolic pathways, Sulfasalazine and P7C3 result in the highest increase in T-cells (Figure 4C). Those agents, along with Minocycline, also resulted in the most elevated CD8-to-FoxP3 ratios, indicating potentially favorable modulation of immune cell responses (Figure 4D). For macrophages, metabolic modulators such as BPTES, Sulfasalazine, SnMP and BSO induced the highest ratio of Stat1-to-Arginase-1 (indicative of M1 polarization) in the brain microenvironment. In contrast, several agents that are more commonly used to treat CNS disorders such as Minocycline, Riluzole and Memantine, exhibit a reversed effect with lower Stat1/Arginase-1 ratios, indicating a potential shift towards M2 polarization as a result of these drug treatments. Macrophage polarization has been linked to neuroinflammation, with M1 state being associated with a pro-inflammatory state (Cherry et al., 20142014; Devanney et al., 2020; Han et al., 2021). The phenotyping provided by the IMD for each of the agents could be an important measurement to understand a novel agent’s therapeutic potential.

## Case study: *In situ* evaluation of 12 drugs as an *in vivo* systems pharmacology approach to treating Alzheimer’s disease

Alzheimer’s disease (AD) is a progressive neurodegenerative disorder involving the accumulation of amyloid-β (Aβ) plaques and tau protein-containing neurofibrillary tangles (NFTs) in conjunction with the acute and chronic inflammation leading to cognitive dysfunction (Lee et al., 2001; Spires-Jones et al., 2009; Zhang et al., 2021). While there are significant knowledge gaps around connecting molecular and cellular phenotypes observed in AD patients to clinical disease progression, specific pathophysiological features of the AD brain have been identified which have become the target of potential therapeutic interventions. For instance, it has become evident that extracellular Aβ plaques and intraneuronal NFTs are associated with neurodegeneration and ultimately memory loss with dementia (Braak et al., 2011; Zhang et al., 2021). NFTs are intracellular aggregates of the microtubule associated protein tau, which is abnormally hyperphosphorylated by upregulation of kinase activity such as GSK-3, and CDK5 or a deficit in phosphatases such as PP2A (Spires-Jones et al., 2009; Badiola et al., 2012; Perea et al., 2020). Neurofibrillary pathology, in particular, has been closely linked to cognitive decline and neurodegeneration, which has generated interest in studying pharmacological interventions targeting tau aggregation. A key question in the field is whether targeting tau phosphorylation through inhibition of protein kinase or phosphatase activation could provide a valid therapeutic approach to reduce tau aggregation, neuronal death, and inflammation, and whether inhibition of Tau phosphorylation would ultimately lead to neuroprotection, neurogenesis, modulation of immune function and anti-inflammatory effects.

To demonstrate how the IMD may be used to rapidly measure such drug effects in the live brain, we performed a case study measuring drug response phenotypes to 12 pharmacological agents across two murine models of AD, a transgenic (TG) htau hemizygous for TG MAPT expression multiple tau isoforms, and non-carrier (non-TG). The drugs tested include commonly used drugs for the treatment of AD, as well as novel compounds including metabolic interventions which have recently been implicated to have potential utility in AD (Table 1).

**TABLE 1:** Overview of drugs and investigational compounds used with IMD platform for multiplex *in situ* drug response screen.

Minocycline	Donepezil	Riluzole	SAHA	ABAH	Paclitaxel
Tetracycline antibiotic	inhibitor of acetylcholinesterase (AChE)	Glutamate inhibitor.NMDA receptor inhibitor	HDAC inhibitor	Myeloperoxidase inhibitor	Microtubule polymer stabilizer
**P7C3**	**Sulfasalazine**	**Etomoxir**	**Buthionine Sulfoximine (BSO)**	**AGI-25696**	**SnMP(Tin mesoporhyrin)**
Neuroprotective chemical that targets NAMPT	Inhibitor of NF-kB,TGF-β and COX-2	Inhibitor of carnitine palmitoyltransferase-1	Depletes cellular glutathione levels	Methionine adenosyltransferase 2A(MATA2) inhibitor	Inhibitor of heme oxidation

### A. Measurement and definition of a drug-specific phenotype for each therapy from a single brain

**FIGURE 5:**
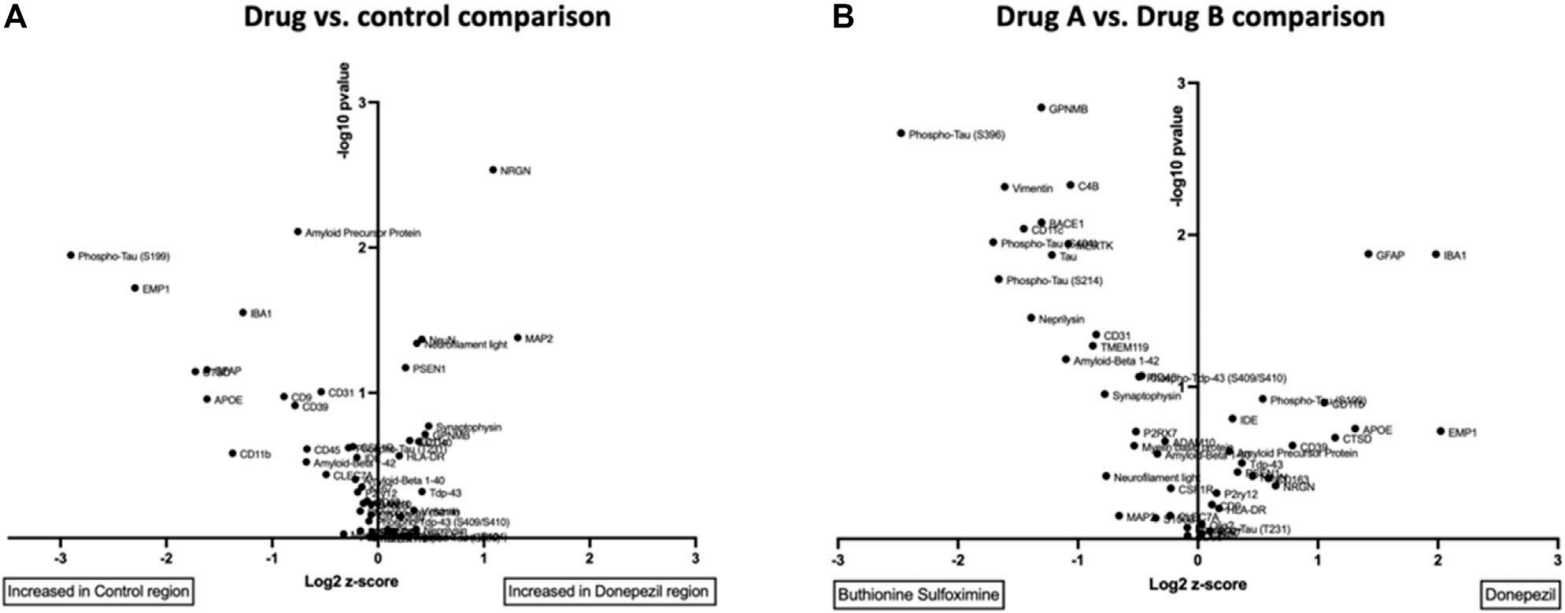
**(A)** Volcano plot comparisons of expression of 56 protein markers in brain region treated with Donepezil vs control in TG model after drug exposure of 7 days **(B)** Direct comparison of marker expression between two drugs, Donepezil versus BSO in TG model. Each unit increase on *x*-axis represents a doubling in marker intensity. The *y*-axis shows the associated *p*-value (-log_10_) across the biological replicate set of n = four to six.

To assess drug responses, we used high-plex spatial proteomics measuring the expression levels of 56 proteins related to brain physiology and AD, including several Tau isoforms. For each of the 12 drug treatments, the tissue response of a region of brain tissue adjacent to the drug delivery reservoir of the IMD (∼400 μm in width) and exposed to a given drug is characterized across all markers. This enables the identification of which biomarkers are most affected by each drug treatment versus an untreated control region (Figure 5A), as well as in a head-to-head comparison between two different drugs (Figure 5B). Twelve drug-versus-control comparisons and 66 inter-drug pairwise comparisons were measured in each mouse. A direct comparison between a drug commonly used clinically in the treatment of AD, donepezil, and an investigational metabolic intervention, buthionine sulfoximine (BSO), reveals the differential effect of each drug on expression of key brain and AD markers. For instance, levels of Tau and various phospho-Tau markers, Neprilysin, as well as CD11c are significantly reduced by donepezil, while IBA-1 and GFAP are reduced in BSO-exposed TG brain regions (Figure 5B).

### A systems-level analysis of functional drug response by probing of AD brain with multiple pharmacological perturbations

**FIGURE 6:**
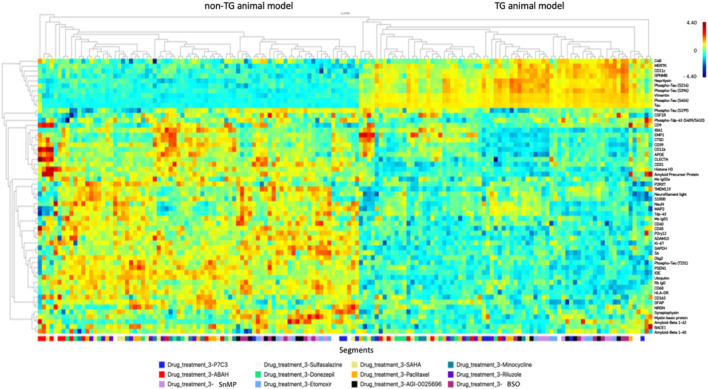
Unsupervised clustering map of drug responses to 12 agents as measured by 56 protein markers, for TG and non-TG mouse models at t = 7 days. Scale is shown in log2 difference.

Figure 6 provides an overview of the effects of 12 drugs across all biomarkers and both mouse models. We performed unsupervised clustering of individual drug responses and observed stark differences in various isoforms of Tau between animal models TG and non-TG, as is expected from model characteristics. We also observe significant GFAP activation and microglia activation in TG model which are associated with neuroinflammation and toxicity, leading to neuronal cell death.

The algorithm clusters regional tissue responses, which are colored to drug. We observe that regions exposed to the same drug tend to cluster together, indicating that consistent response phenotypes emerge for each agent. Furthermore, drug regions with similar response profiles cluster together, thus indicating a high degree of concordance across the range of protein expression markers employed. For instance, BSO and Etomoxir, two compounds affecting mitochondrial metabolism, exhibit a high degree of clustering, which indicates largely similar effects on brain tissue across markers. Specific phenotypes associated with favorable effects on AD-associated markers are described in the following section.

**FIGURE 7:**
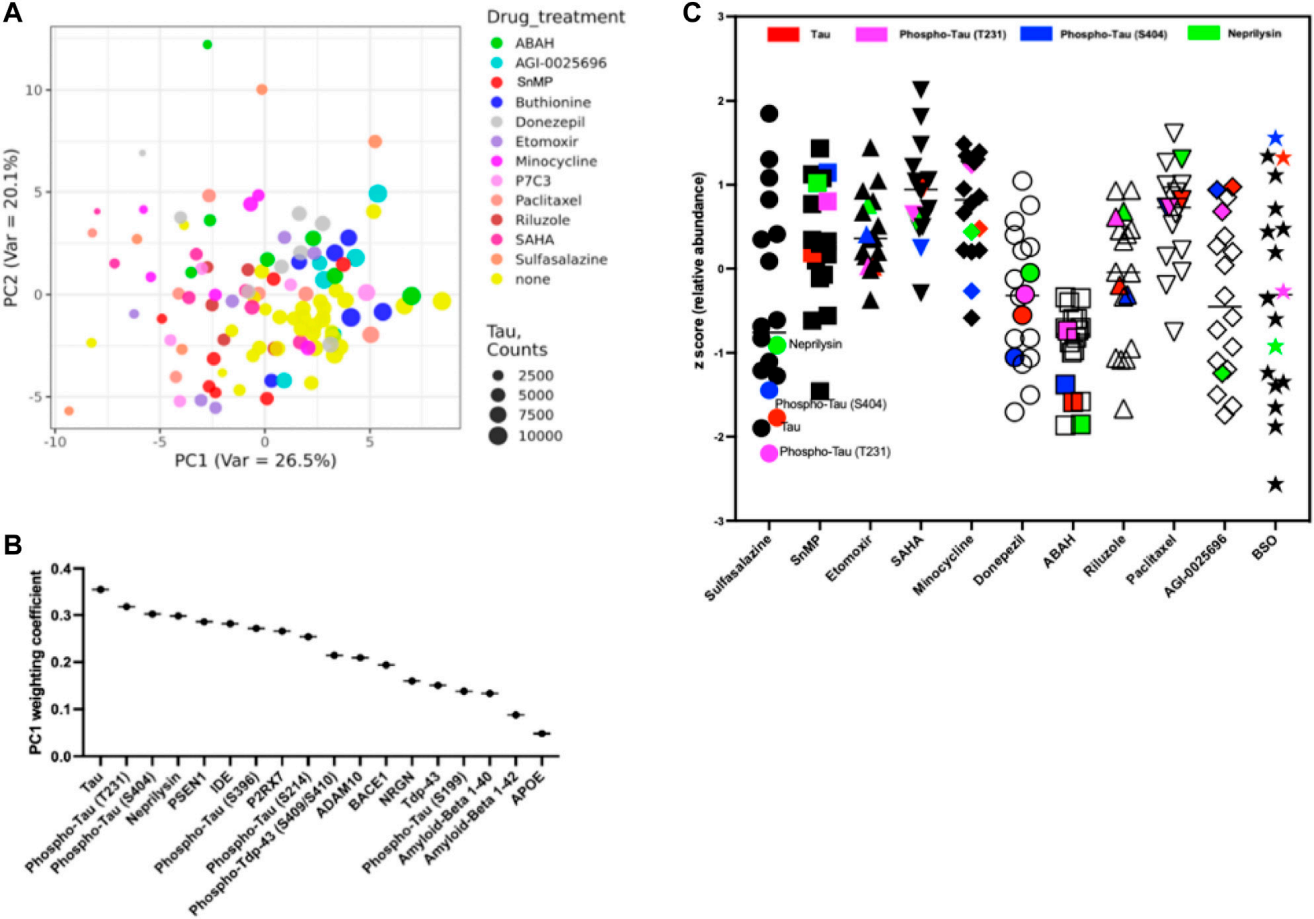
**(A)** Principal component analysis reveals distinct phenotype of drug activity at t = 7 days **(B)** Identification of most significantly affected individual markers from principal component from all drugs tested in TG model **(C)** Drug effects in reduction of AD-associated markers (z-score). Highlighted are key markers of tauopathy (Tau: red; phospho-Tau T231: magenta; phospho-Tau T404: blue; and Neprilysin: green).

A. Biomarker discovery and definition of response phenotype for anti-AD drugs:

We performed principal component analysis on the normalized expression data to identify protein markers that are characteristic of drug responses in the TG model (Figure 7A). The dominant principal component weightings indicate which of the 56 markers employed are most affected by the set of 12 drugs versus the control. We observe that the primary principal component, accounting for 29.4% of variability, has dominant loadings in total Tau, two Phospho-Tau isoforms (T231 and S404) and Neprilysin. The relative weightings are shown in Figure 7B. Markers with the highest weighting coefficients are determined to be most affected by the set of drugs being used.

B. Identification of most effective treatments at effecting a desired phenotype in hMAPT TG brain:

A key question in the development of effective therapies for the treatment of AD is whether a given therapy induces favorable pathobiological changes in the affected tissue. Using the markers associated with AD response defined in Figure 6, we determined that among the drugs tested, ABAH had the greatest effect on reduction of markers associated with AD, with consistent reductions across all markers. Among the investigational metabolic interventions, Sulfasalazine (inhibitor of NF-κB, TGF-β and COX-2) elicits the most favorable response profile, though the effects show greater variability and are most prominent in markers for Tau family proteins (Figure 7C).

## Discussion

Here, we describe a novel platform for high-throughput *in-situ* drug response measurements in the mouse brain. The approach enables researchers to simultaneously deliver microdoses of multiple drugs, metabolites, tool compounds or probes directly into specific regions of the brain, and characterize the effects of each compound on native brain tissue in a concentration-dependent manner using a variety of spatial analysis techniques. Systems level analyses of up to 16 independent pharmacological perturbations are performed in the same organism and within the native brain microenvironment.

Whereas in traditional systemic *in vivo* studies, only one compound or combination can be measured per animal, the IMD approach enables up to 16 per mouse brain. This enables the measurement of multiple *in vivo* drug response phenotypes without the need to optimize a compound for systemic PK or toxicity. This may be especially valuable for tool compounds that are not optimized for penetration of the blood-brain barrier. The IMD approach also presents significant advantages versus *ex vivo* and *in vitro* systems. Such systems typically only include a subset of cell types present in the intact brain, and thus cannot replicate natural cell-cell interactions integral to disease pathology. They also do not faithfully recreate three-dimensional brain architecture, which is critical for such cell-cell interactions, and generally exclude the effects of T-cells and other immune cells which play important roles in inflammatory neurodegenerative disorders.

This pilot study demonstrates precise localized release of multiple drugs into confined regions of the brain. In previous cancer studies using the IMD [ (Jonas et al., 2015)], we have demonstrated that the molecular weight of the formulation polymer is the primary determinant of drug diffusion, and that consistent diffusion gradients are obtained for a range of small-molecule drugs across the range of ∼250–800Da for different drug solubilities. A limitation of the current study is that it only demonstrates direct measurement of the localized drug release for autofluorescent compounds (Figure 2). It is thus conceivable that differences in local concentration may have contributed to variability in PD effects. MALDI mass spectrometry tissue imaging is a technique that could be used to detect the intra-brain release of non-fluorescent compounds [(Davidson et al., 2016; Jonas et al., 2016)], though it requires sophisticated instrumentation and user expertise.

Our pilot study shows compound specific effects on AD pathophysiology that are consistent with favorable anti-AD effects in brain tissue. For instance, we show that several treatments, most notably ABAH and Sulfasalazine, lead to a significant reduction in activated astrocytes as measured by GFAP, and a reduction in Iba-1 expression, 7 days after microdevice implantation. These agents also caused a significant reduction in levels of Tau, phospho-Tau and Neprilysin expression in the AD brain. These four markers were identified by principal component analysis to be the most significant in determining a favorable drug response phenotype. Tau phosphorylation is closely associated AD pathological processing and many phosphorylated sites are known as Ser199-202-Thr 205 (AT8), T 231, and Ser 396-404 (Paired Helical Filaments) during NFT formation in AD pathology (Gu et al., 2013; Mondragón-Rodríguez et al., 2014). Neprilysin is a metallopeptidase which functions as an amyloid-degrading enzyme, has been shown to alter the neuropathological and behavioral phenotype in the 5XFAD mouse model of AD (Nalivaeva et al., 2020), and is associated with cognitive function (Hüttenrauch et al., 2015). We also demonstrated differential effects of agents on the T-cell and macrophage population in the AD brain. The IMD approach is capable of identifying compounds (and local concentrations) that are more effective at inducing specific T-cell activation or macrophage polarization states. These findings demonstrate significant potential for the IMD approach to be used for discovery and validation of biomarkers of drug response.

It should be noted that due to the small size of the mouse brain, a precise targeting of drug release exclusively into the dentate gyrus (DG) may not always be feasible, and it is likely that areas of drug release span both the DG and thalamus. This may be overcome by directly targeting larger structural regions of the mouse brain such as the cortex or thalamus. If the DG is to be targeted exclusively, larger species such as rats may be more amenable.

We determined that the set of agents tested also have markedly increased effects in the TG versus non-TG and WT brains, indicating a degree of specificity towards the AD phenotype. The ability to test such a large set of compounds in a single model allowed us to use a systems approach to identify the markers most affected by the set of AD drugs and investigational compounds. Such an approach can be used in comparative studies involving standard of care and investigational treatments to better perform functional characterization of disease state in a given organism or model. It is important to note that the system is agnostic to the markers being employed, and users are free to apply other IHC/IF markers as a readout of drug activity or immune or microenvironment response.

Limitations of the IMD approach for prioritizing systemic treatment regimens are primarily two-fold. One, the approach does not take into account varying systemic PK properties of compounds, especially the ability to penetrate the BBB, as the compounds are delivered directly into the brain from reservoirs, and not through the systemic vasculature. While this allows for much earlier and less toxic testing of compounds directly in the brain, BBB penetration is not directly measurable with this approach, and will likely continue to require systemic dosing studies. If a given drug is found to be effective via the IMD at higher concentrations than would be achievable with standard systemic administration, alternative means may be required by which an effective drug could be delivered in sufficient concentrations to the brain (Patel et al., 2009; Patel et al., 2009; Gabathuler, 2010). Examples of such approaches include convection-enhanced delivery (Mehta et al., 2017), disruption of the BBB (e.g. by focused ultrasound) (Aryal et al., 2014), chemical or nanocarrier delivery systems (Finbloom et al., 2018), and the use of intracranial drug-eluting implants (Gazaille et al., 2021), among others. In such a scenario, the IMD may be used to identify the required concentration to induce a desired biological effect in the diseased tissue, and the clinician would choose an appropriate enhanced delivery technique in an attempt to achieve an effective local dose. In order to make such PK data from the IMD actionable in patients, additional studies will be needed in humans to establish the relationship between drug concentrations in the serum and those in the brain tissue, following systemic treatment.

Secondly, the measured drug phenotypes are near-term, local pathobiological measurements, and therefore will not capture systemic effects or longer-term cognitive behavior changes such as memory loss. Such studies investigating longer-term behavioral modification will likely continue to rely on systemic administration of test compounds, but the IMD platform can help prioritize among large sets of candidate compounds based on *in situ* pathobiological drug effects, and can furthermore provide high-throughput correlation of near-term phenotypic effects of drugs on disease biology, with longer term outcomes.

### Future Directions and Outlook

Though the current study demonstrates application in the mouse brain, we expect that analogous studies can readily be conducted in other animal species such as rats or non-human primates. Larger brains would allow for insertion of longer IMDs with more reservoirs, as well as multiple IMDs per brain. In the current study we demonstrate up to 16 drug responses measured per mouse brain, but in rats it may be feasible to obtain 30–50 drug responses and in primates up to 100 or more drug phenotypes per brain.

We envision that the technique may also be used to measure the effect of novel therapeutic agents and combinations directly in patients’ brains, where such measurements could be performed in parallel to already scheduled biopsies or other brain interventional procedures to obtain *in situ* functional drug readouts, for instance in brain tumors, CNS vasculitis, or during placement of deep brain stimulation electrodes. This is already being performed in a currently ongoing clinical study of the IMD in brain tumor patients (NCT04135807). For broader applicability, an IMD prototype incorporating optical readouts (Fluorescence and Raman spectroscopy) of drug effects is currently under development and has been demonstrated in oncology applications (Jonas et al., 2018; Bhagavatula et al., 2021). In CNS studies, this advance would obviate the need for tissue removal from the patient to enable multiplexed real-time drug effect analysis in live animals or patients.

## Online Methods

### Structure, size, material and manufacturing of the IMD

The IMD prototype used in the current study is optimized for use in the brain. It is a cylindrical construct of 4 mm length and 620 um diameter that is micromachined on a 5-axis CNC mill (MDA Precision) from radiopaque poly-ether-ketone-ketone (PEKK) polymer (Oxford Performance Materials), and fits inside a conventional 22 g biopsy needle. There are between 8 and 20 micro-reservoirs on the device mantle. Cylindrical reservoirs are drilled on the outer surface of IMDs in dimensions of 200 μm (diameter) × 200 μm (depth). Adjacent reservoirs were positioned 800 μm apart to prevent the intersection of compounds in tissue.

1-2 IMD can be inserted into the mouse brain. A pointed tip minimizes tissue damage during insertion. A flat tab at the end of the IMD facilitates attachment to a stereotactic frame during insertion into the brain.

To facilitate external use of the technology, the authors agree to provide 3D models and machining code files to academic collaborators interested in making IMDs in their laboratory. Requests from commercial entities will be evaluated on a case-by-case basis.

### Drugs and Compounds

Drugs for this study were purchased from Selleck Chemicals (Minocycline, Vorinostat (SAHA), Donepezil, 4-aminobenzoic hydrazide (ABAH), Riluzole, Paclitaxel, Sulfasalazine, SnMP, P7C3, Etomoxir, BSO, and MK2206). AGI-25696 was purchased from Adooq Bioscience (A20250). AD drugs are chosen based on differential target function for Alzheimer’s disease or specific metabolic pathway (Table 1). Drugs were formulated with polyethylene glycol (PEG) 3,400 or 8,000 (Spectrum Chemical) as described in (Jonas et al., 2015). Briefly, compounds in the ratio of 20% drug were added to 80% PEG-3450 or PEG-8000 and vortexed for 5 min above its melting point (37 °C). For insoluble drugs, a mixture of drug, PEG, and an organic solvent (ethanol or acetone) was heated to ∼37°C until completely dissolved. The solution was placed on a rotary evaporator (Buchi) for ∼30–40 min at below respective vapor pressures to completely evaporate the solvent, leaving a homogeneous solid mixture of drug and PEG.

Drug/polymer mixtures were loaded into micro-reservoirs as described in (Jonas et al., 2015). Local drug concentration is measured using drug autofluorescence or Maldi imaging mass spectrometry as described in (Jonas et al., 2015; Davidson et al., 2016; Jonas et al., 2016), and calibrated using homogenized drug-tissue sections of identical thickness.

### Animal Models of Alzheimer’s Disease

Three animal models were used in this study: 1. “TG”: B6. Cg-Mapt tm1(EGFP)Kit Tg (MAPT) 8cPdav/J (Stock No: 005491/htau hemizygous for Tg MAPT) 8cPdav; 2: “non-TG”: Non-Carrier for Tg (MAPT) 8cPdav; and 3: “WT”: C57black/6 mice; all mice were purchased from Jackson Laboratories. Human Tau mice were originally generated by Peter Davis using human Tau transgene (Andorfer et al., 2003). The mice used in this study contain the microtubule associated protein human Tau (hMAPT) and express all six isoforms (including 3 and 4R forms) of hMAPT. Male mice aged 12–16 weeks were used for this study. Food and water were freely accessible to the mice, and the holding rooms were maintained on a 12-h light/dark cycle.

### Intracranial Insertion of IMD

The IMD placement procedure is analogous to stereotactic injections routinely performed in CNS research. Stereotaxic surgery, as previously described (Kim et al., 2009), is used for brain microdevice implantation. Mice are anesthetized with 1.5% isoflurane, 50% N_2_O, and 30% O_2_ inhalation, and then placed in a stereotaxic surgical apparatus (David Kopf Instruments, United States). Specific regions of the brain are targeted by proper positioning of the mouse on the stereotaxic frame, and selection of desired coordinates of the targeted brain region from a mouse brain atlas. The IMD is held by its rear tab by the clamp on a standard stereotaxic surgical apparatus. The IMD is inserted either directly into the brain at the desired position, or via a biopsy needle where it is pushed through the needle by a stylet until fully inserted at the desired depth. A Hamilton syringe (Hamilton, United States) needle is attached into the left bregma for dentate gyrus(DG) for coordinates from the bregma: anterior–posterior (2.1 mm), medio-lateral (1.5 mm), and dorsoventral-ventral (4.0 mm); Drug loaded microdevices are implanted in dentate gyrus (DG) and remain *in situ* for three or 7 days after implant. Treatment groups consisted of 1) sham-operated (Sham surgery, no device implant); 2) Wild type mice (C57/Black, microdevice drug load). 3) Non-carrier MAPT mice (Non-TG) 4) MAPT Transgenic AD mice (TG). Each group comprised five animals per experiment.

Though we targeted IMD placement into the dentate gyrus (DG), given its small size in the mouse brain, it is likely that drug diffusion extended beyond the DG and occurred predominantly into the thalamus.

All animal procedures were conducted in accordance with protocols approved by the BWH Institutional Care and Use Committee and following National Institutes of Health guidelines on the care and use of animals.

### Brain Processing, Embedding and Sectioning

Mice are euthanized by CO_2_ exposure and transcardially perfused with phosphate-buffered saline (PBS; pH7.4). Brain Tissues are immersed in 4% paraformaldehyde for 48 h and placed into 70% EtOH before standard paraffin processing.

The excised brain is fixed, processed and sectioned with the IMD remaining in place.

Multiple paraffin-processed brain samples are embedded in a paraffin block and sectioned on a standard microtome. At the first reservoir level of the IMD (identifiable by visual confirmation) serial sections of 4 micron thickness are collected. Sectioning continues until the next IMD reservoir level is reached, where more sections are collected. This process continues until sections from all IMD levels are obtained.

The spatial location of the IMD drug delivery reservoirs to the adjacent tissue is confirmed for each specimen by including at least one autofluorescent compound (drug or tool compound or probe) in a reservoir of the IMD. When the brain containing the IMD is sectioned at the reservoir level containing the autofluorescent drug, the tissue section is imaged. When the fluorescent signal of the drug is observed at the correct depth and rotational orientation of the reservoir, then it is confirmed that the IMD has not rotated or moved longitudinally.

### Multi-Color Immunofluorescence Staining and Imaging

Prior to staining, antigen retrieval/Quench (Leica Biosystem, RE7113-CE, PH6 or PH9) was performed in microwave for 10–30 min (Microwave research and applications Inc. Model: BP-093). Brain sections are deparaffinized in dry incubator for 30 min at 60 °C (BOEKEL Scientific) and subsequently immersed with xylene solution following gradient ETOH solution for 5 min. The brain sections are incubated with primary antibodies for 30 min at room temperature. Brain sections are washed three times with TBST solutions and incubated with Novacastra polymer (Leica Biosystem, Cat. # RE7101-CE) for 10 min. After that, sections are washed with TBST buffer and peroxidase blocking solution is applied (Leica Biosystem, Cat# RE7101-CE). For multi-color staining, we incubated Opal fluorophore for 5 min (1:50 dilution) (stock Opal Flour is diluted 1:75 in DMSO).

Slides were then rinsed with TBST buffer, followed by antigen retrieval/quench in microwave for multi-color staining for 10–30 min. Continuously, slides were treated with other antibodies and rinsed in DI water for 20 min. After that, the brain sections were mounted with diamond mounting media and placed on an automated fluorescent slide scanner (Leica Aperio Versa) for imaging.

### Spatial proteomics

The GeoMx platform (Nanostring, Seattle United States) was utilized to quantify spatial protein expression for 56 protein markers from FFPE brain tissue sections. After preparation of the sections (baking, antigen retrieval, blocking), a multi-plex cocktail of profiling antibodies barcoded with photocleavable and uniquely indexed oligonucleotides were incubated on the tissues. The sections were additionally stained with a nuclear marker Syto13 for downstream fluorescent visualization on the GeoMx instrument via the FITC channel.

Regions of interest (ROIs) on FFPE slides were selected based on adjacency to drug delivery reservoirs in increments of 200 microns. Additionally, control ROIs were selected adjacent to a part of the microdevice with no drug reservoir. After ROI selection, the GeoMx was used to UV-photocleave and collect oligonucleotides from the profiling antibodies staining the tissue, which were then deposited into a 96 well collection plate. Each ROI was illuminated independently to enable spatially resolved data acquisition. Oligonucleotides were then hybridized to fluorescent barcodes and quantified with the nCounter^®^ (Nanostring) Analysis System. The raw digital counts for the oligonucleotides were calibrated for oligo-barcode binding by the GeoMx. The data was then normalized to spike-in positive controls to assess data quality. The calibrated and spike-in normalized expression data was then normalized at a per ROI level to the geometric mean of the housekeeping controls (S6, GAPDH) to enable the study of downstream differential expression.

### Statistical Analysis

The data were analyzed using Prism 6 (GraphPad, United States). Data were reported as the mean ± S.E.M. The number of mice used for each group was calculated to achieve a power of 90% resulting in n = four to six per group. In Figure 5, we performed a non-paired *t*-test to generate the *p*-values, which were then plotted in a volcano plot. To generate the clustering plot shown in Figure 6, we performed unsupervised standard Euclidean clustering on the normalized protein expression data (Figure 6) as described in (Hastie et al., 2009). The principal component analysis (PCA) in Figure 7A,B was performed as described in (Jolliffe, 2002).

## Data Availability

The original contributions presented in the study are included in the article/Supplementary Files, further inquiries can be directed to the corresponding author/s.
