# Supplementary material for: Intratarget Microdosing for Deep Phenotyping of Multiple Drug Effects in the Live Brain
Source: Front Bioeng Biotechnol. 2022 Mar 18;10:855755. doi: 10.3389/fbioe.2022.855755 (PMC8973214; doi:10.3389/fbioe.2022.855755)
Supplement: Supplementary file 1 [file DataSheet1.pdf]

SUPPLEMENTAL FIGURES:

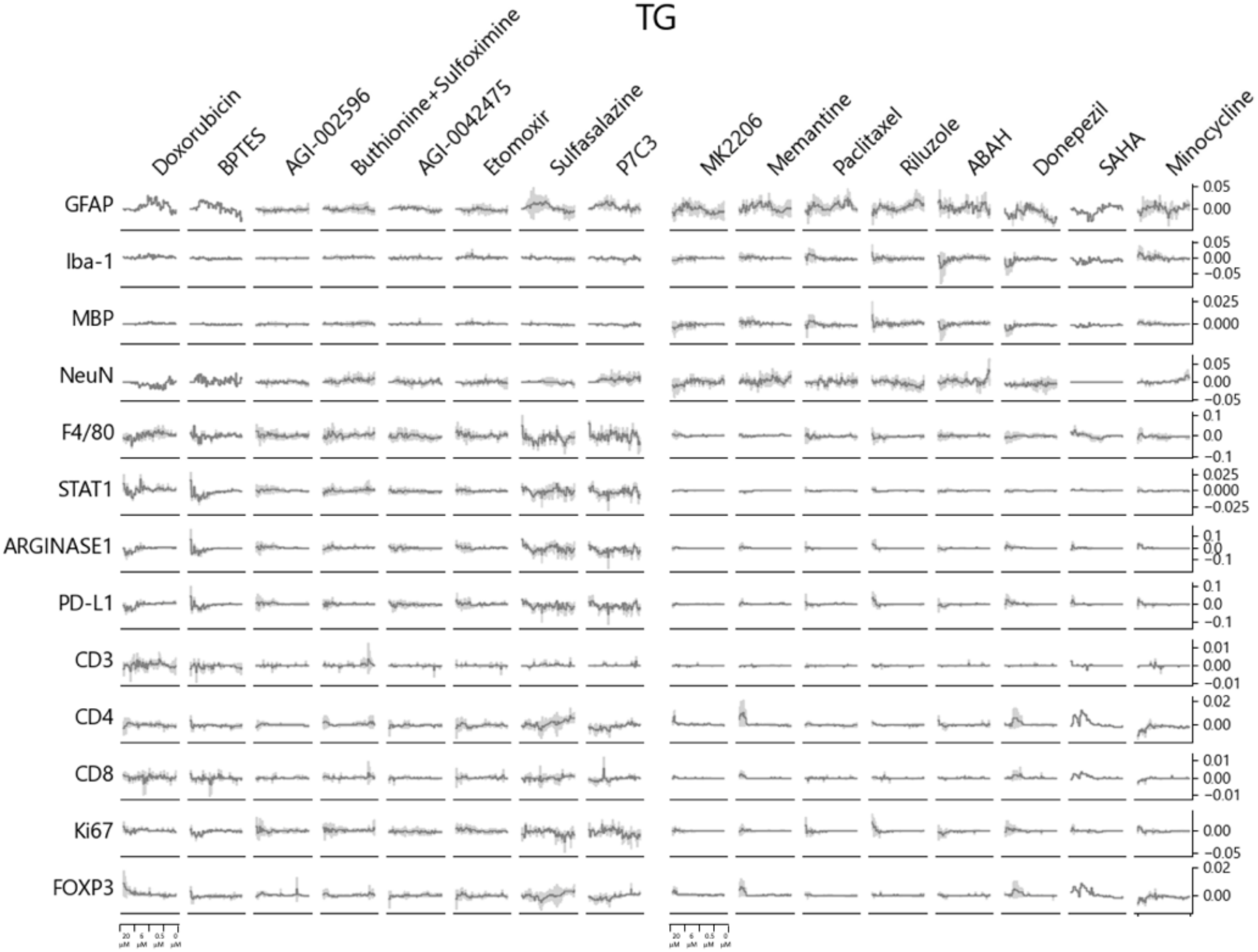

Supplemental Figure 1: Overview of PK/PD curves for 16 pharmacological agents in the TG mice across 13 biomarkers. All curves display fluorescent intensity for a given IF marker, and are averaged from 4-6 ROIs per drug. Values represent the net change of marker expression versus the control expression level.

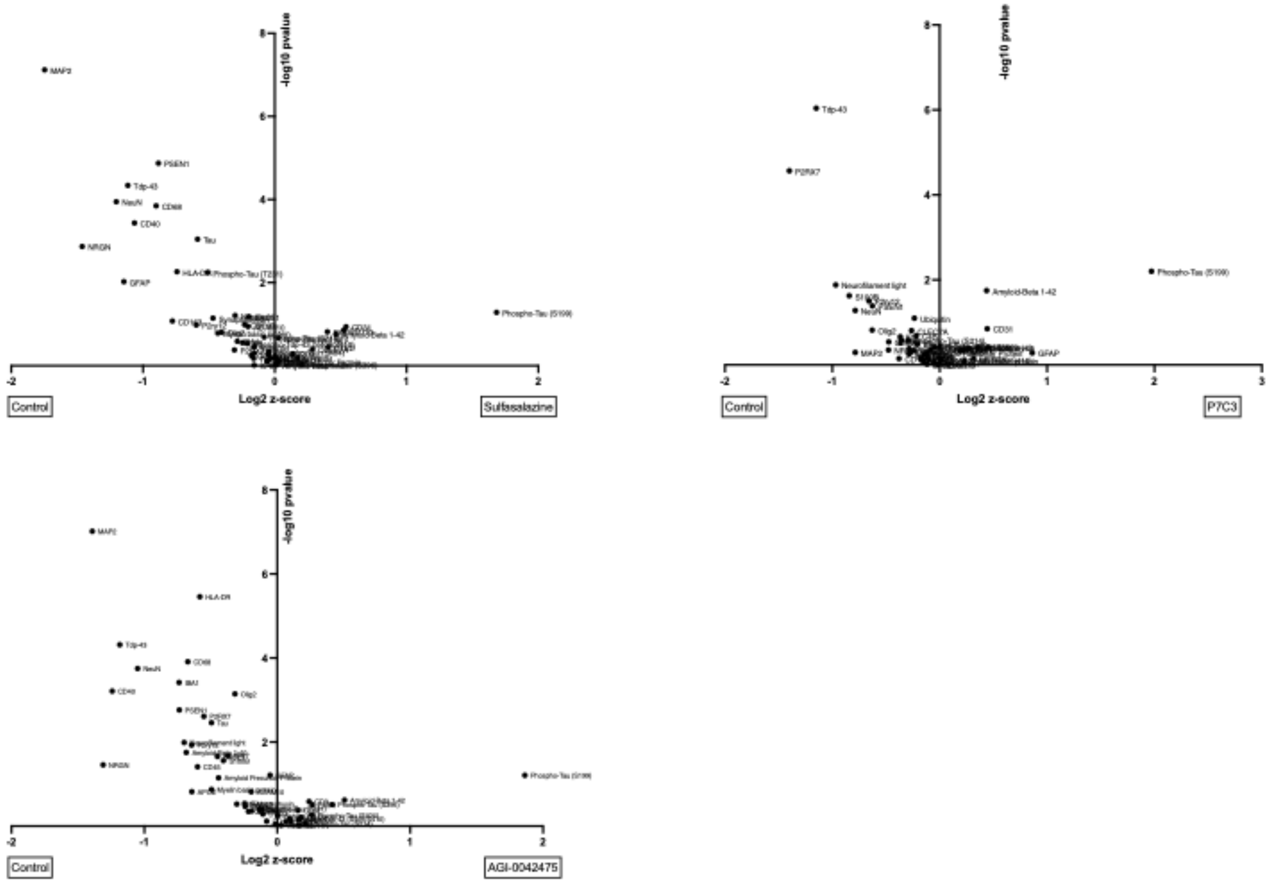

Supplemental Figure 2: Additional volcano plots showing differential protein expression in drug and control tissue regions. Each unit increase on x-axis represents a doubling in marker intensity. The y-axis shows the associated p-value ($-\log_{10}$) across the biological replicate set of n=4-6.
